# Hypercalcemic Crisis in Pregnancy With Acute Respiratory Distress Syndrome Requiring Extracorporeal Membrane Oxygenation

**DOI:** 10.1210/jcemcr/luaf191

**Published:** 2025-08-22

**Authors:** Shinnosuke Hata, Shunya Tanaka, Naoki Ehara, Masahito Horiguchi, Tsuneyuki Nakanouchi, Toru Tanaka

**Affiliations:** Department of Endocrinology and Metabolism, Japanese Red Cross Society Kyoto Daiichi Hospital, Kyoto 605-0981, Japan; Department of Endocrinology and Metabolism, Kyoto Prefectural University of Medicine, Graduate School of Medical Science, Kyoto 602-8566, Japan; Department of Respiratory Medicine, Japanese Red Cross Kyoto Society Daiichi Hospital, Kyoto 605-0981, Japan; Department of Emergency and Critical Care Medicine, Japanese Red Cross Society Kyoto Daiichi Hospital, Kyoto 605-0981, Japan; Department of Emergency and Critical Care Medicine, Japanese Red Cross Society Kyoto Daiichi Hospital, Kyoto 605-0981, Japan; Department of Nephrology, Japanese Red Cross Society Kyoto Daiichi Hospital, Kyoto 605-0981, Japan; Department of Endocrinology and Metabolism, Japanese Red Cross Society Kyoto Daiichi Hospital, Kyoto 605-0981, Japan

**Keywords:** acute respiratory distress syndrome, hypercalcemic crisis, parathyroid storm, primary hyperparathyroidism

## Abstract

Primary hyperparathyroidism (pHPT) during pregnancy is a rare but clinically significant condition associated with severe maternal and fetal complications. Diagnosis and management are challenging owing to symptoms overlapping with pregnancy-related conditions. A 28-year-old woman at 6 weeks of gestation presented with severe hypercalcemia (ionized calcium 2.80 mmol/L) (reference range, 1.15-1.29 mmol/L) leading to acute respiratory distress syndrome (ARDS), renal failure, and fetal demise. Despite initial management with hemodialysis and veno-venous extracorporeal membrane oxygenation (V-V ECMO), hypercalcemia and impaired respiratory status persisted until a parathyroid nodule was resected surgically. Postoperatively, calcium levels normalized, and the patient was successfully weaned off V-V ECMO; however, chronic kidney disease persisted. This case highlights the systemic effects of severe hypercalcemia in pregnancy, including ARDS and nephrocalcinosis. The case underscores the importance of a timely diagnosis and surgical intervention, as well as the need for a multidisciplinary approach to optimize outcomes in complex cases of pHPT during pregnancy. This report emphasizes the critical role of early recognition and management of pHPT during pregnancy to mitigate severe complications. Future research should focus on improving diagnostic strategies and refining treatment protocols for complex cases involving hypercalcemia-induced multiorgan dysfunction.

## Introduction

Primary hyperparathyroidism (pHPT) during pregnancy is an uncommon but clinically significant condition, posing potential risks both to maternal and fetal health [1]. Diagnosis of pHPT is challenging owing to the nonspecific nature of its symptoms, which often overlap with those of common pregnancy-related disorders [2]. Maternal complications associated with pHPT include nephrolithiasis, pancreatitis, and hypercalcemic crisis, while fetal complications can manifest as neonatal hypocalcemia, stillbirth, and neonatal death [2]. The incidence of fetal complications in mothers with untreated pHPT has been reported to reach up to 80% [1].

Management of pHPT in pregnancy is particularly challenging owing to the limited safety profile of medical treatment options. Meticulous monitoring of maternal serum calcium levels is essential to optimize outcomes both for mother and fetus [3].

Herein, we present a previously unreported and rare case of pHPT requiring veno-venous extracorporeal membrane oxygenation (V-V ECMO) in pregnancy to contribute to the growing body of knowledge and enhance understanding the clinical management of pHPT.

## Case Presentation

**Figure 1.**
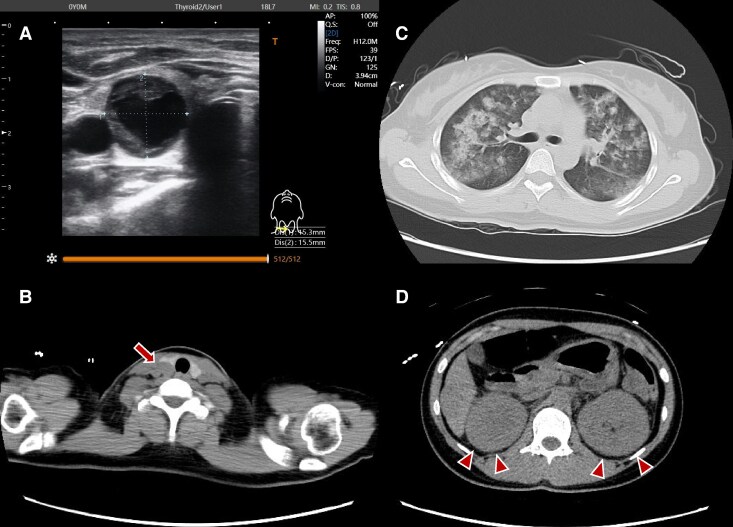
Imaging of the neck, thorax, and abdomen. A, Cervical ultrasonography showing a nodule near the lower pole of the right thyroid lobe. B, Cervical computed tomography (CT) imaging showing a 20-mm mass in the same region (arrow). C, Chest CT showing alveolar opacities, predominantly in the upper lung fields bilaterally. D, Abdominal CT images consistent with acute kidney injury, characterized with bilateral renal enlargement (arrowhead).

A previously healthy 28-year-old woman at 6 weeks and 6 days of gestation presented to her attending obstetrician with complaints of lower abdominal pain and vomiting. She was in her second pregnancy, with one prior live birth. The patient was referred to our hospital 2 days prior to the 6-day New Year holidays in Japan with right lower abdominal pain. The patient's clinical parameters were as follows: height, 163.0 cm; weight, 49.0 kg; blood pressure, 131/92 mm Hg; heart rate, 83 beats/min; body temperature, 37.0 °C; respiratory rate, 20 breaths/min; and SpO_2_, 99% (room air). Her respiratory status worsened and respiratory failure developed on day 2. Coarse crackles were heard on pulmonary osculation, heart sounds were regular without murmurs, and no edema was observed on the extremities. In light of the worsening respiratory condition, prioritization of maternal survival led the obstetrician to perform a computed tomography (CT) scan. The cervical-to-pelvic CT scan showed bilateral renal enlargement, bilateral alveolar opacities primarily in the upper lung lobes, and a 20-mm-hypodense nodule in the right lower pole of the thyroid gland (Fig. 1). These findings were suggestive of acute renal injury (AKI), pulmonary edema, and a parathyroid nodule, respectively. Laboratory tests showed high ionized calcium levels (2.80 mmol/L) (reference range, 1.15-1.29 mmol/L) and an estimated glomerular filtration rate (eGFR) of 22 mL/min/1.73 m^2^ (reference range, ≥60 mL/min/1.73 m^2^). An electrocardiogram (ECG) showed shortening of the QT interval, but neither ECG nor echocardiography indicated findings of myocardial infarction or heart failure. The patient was referred to intensive care specialists and admitted to the intensive care unit. She had no family history of parathyroid diseases. Given the pregnancy and complications of her hypercalcemia, options for medical treatment were limited. Thus, hemodialysis was initiated. The patient subsequently developed acute respiratory distress syndrome (ARDS) at fraction of inspired oxygen (F_I_O_2_) of 0.55 (P/F ratio: 119) and required mechanical ventilation. Steroid therapy was also initiated. On day 3, the fetal heartbeat was no longer detectable leading to a diagnosis of a missed miscarriage. Hypercalcemia management included the administration of zoledronic acid and evocalcet with hemodialysis due to the inability to continue the pregnancy, resulting in a gradual decline in calcium levels. However, her respiratory condition did not improve owing to persistent ARDS, necessitating the initiation of veno-venous extracorporeal membrane oxygenation (V-V ECMO) on day 4.

## Diagnostic Assessment

Ionized, total, and corrected calcium levels were 2.80 mmol/L, 19.0 mg/dL (SI: 4.75 mmol/L) (reference range, 8.4-10.0 mg/dL [SI: 2.10-2.50 mmol/L]), and 20.7 mg/dL (SI: 5.18 mmol/L) (reference range, 8.7-10.3 mg/dL [SI: 2.17-2.57 mmol/L]), respectively. The urine Ca/Cr ratio was 0.24 mmol/mmol, which was not suggestive of hypocalciuria. The value of inorganic phosphate (IP) was high (4.9 mg/dL [SI: 1.58 mmol/L]) (reference range, 2.5-4.5 mg/dL [SI: 0.81-1.45 mmol/L]), which was atypical for pHPT. Her serum creatinine was 3.02 mg/dL (SI: 267 µmol/L) (reference range, 0.6-1.1 mg/dL [SI: 53-97 µmol/L]), and eGFR was 22 mL/min/1.73 m^2^. Therefore, impaired phosphate suppression attributable to renal dysfunction was considered a possible contributing factor. Since she was transferred just before the beginning of the long holiday period in Japan, intact parathyroid hormone (i-PTH) test results were delayed. 99mTc-methoxyisobutylisonitrile (99mTc-MIBI) scintigraphy could not be performed owing to staff shortages and a lack of testing agents over the holiday period, the instability of her condition, and the uncertain effect of ECMO on image quality. The bronchoalveolar lavage fluid obtained on day 3 did not indicate the presence of alveolar hemorrhage or microliths.

## Treatment

**Figure 2.**
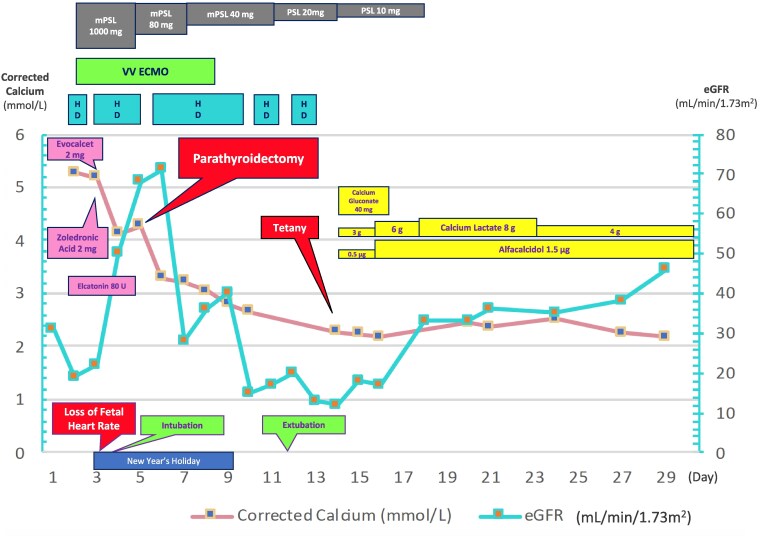
Trends in corrected calcium levels and eGFR alongside the treatment timeline. Following parathyroidectomy, corrected calcium levels returned to normal. On day 14, the patient developed tetany, which improved with the initiation of calcium and vitamin D supplementation. The numbers following the medication names represent the daily dosages. Abbreviations: eGFR, estimated glomerular filtration rate; HD, hemodialysis; mPSL, methylprednisolone; PSL, prednisolone; V-V ECMO, veno-venous extracorporeal membrane oxygenation.

Given the persistent hypercalcemia accompanied by respiratory failure, and the neck image findings, the likelihood of pHPT as a differential was assessed as high, although IP was not suppressed. Because of a lack of improvement in the patient’s respiratory status, a parathyroidectomy was performed on day 6. Postoperatively, serum calcium levels decreased, and both renal and respiratory function improved. Hemodialysis was discontinued on day 9. The patient was successfully weaned from V-V ECMO, and liberated from mechanical ventilation on day 10 (Fig. 2). On day 10, the i-PTH level prior to the parathyroidectomy was found to be high (936 pg/mL [SI: 936 ng/L]) (reference range, 10-65 pg/mL [SI: 10-65 ng/L]) (Table 1).

**Table 1.** Laboratory findings revealed after parathyroidectomy (and reference ranges)

Laboratory tests	Value (in SI units)	Reference range (in SI units)
i-PTH	936 pg/mL (936 ng/L)	10-65 pg/mL (10-65 ng/L)
PTHrP	<1.0 pmol/L	<1.0 pmol/L
25(OH)D	<4.0 ng/mL (<9.984 nmol/L)	30-100 ng/mL (75-250 nmol/L)
1,25(OH)_2_D	29.7 pg/mL (77.22 pmol/L)	20-60 pg/mL (48-144 pmol/L)
ACE	10.3 U/L	8-21 U/L

Despite the presence of hypercalcemia, i-PTH levels were elevated, indicative of primary hyperparathyroidism (pHPT), while PTHrP, 1,25(OH)_2_D, and ACE levels remained within normal ranges.

Abbreviations: 25(OH)D, 25-hydroxyvitamin D; 1,25(OH)_2_D, 1,25-dihydroxyvitamin D; ACE, angiotensin-converting enzyme; i-PTH, intact parathyroid hormone; PTHrP, parathyroid hormone–related protein.

## Outcome and Follow-up

**Figure 3.**
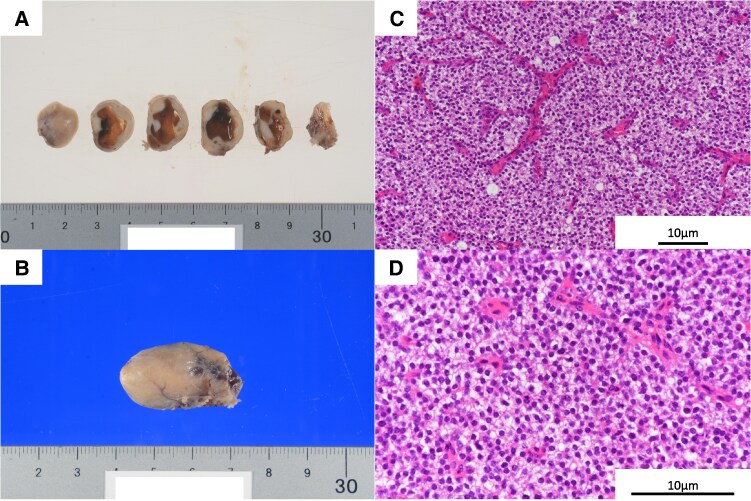
Resected specimen of a right inferior parathyroid gland. A and B, Macroscopically, a solid lesion with cystic components is observed. C, Low-magnification and D, high-magnification hematoxylin and eosin–stained images of the parathyroid nodule. Histologically, the tumor is encapsulated within a thin fibrous capsule. The lesion exhibits a proliferative pattern characterized with nests of cells with clear cytoplasm and round-to-oval nuclei, accompanied with numerous blood vessels.

The pathology of the right lower parathyroid gland revealed a parathyroid adenoma pattern on histopathology. The tumor, consisting of nests of cells with clear cytoplasm and round-to-oval nuclei, was encapsulated within a thin fibrous capsule and contained numerous blood vessels. There was no evidence of vascular invasion or infiltration into the surrounding tissues (Fig. 3). Postoperative calcium levels remained within the normal range, but tetany developed 8 days after surgery. She was treated with a combination of calcium and vitamin D preparations, which were tapered over several months. The patient showed some renal function recovery after AKI during hospitalization; however, she subsequently progressed to chronic kidney disease (CKD). At the last outpatient department visit almost 1 year since discharge, the eGFR was 57 mL/min/1.73 m^2^. The gene panel test for hyperparathyroidism, including *MEN1*, *CDKN1B*, *RET, CASR*, *GNA11*, *AP2S1*, *CDC73*, and *GCM2* showed no special findings.

## Discussion

pHPT during pregnancy is uncommon but carries substantial risks, with overlapping pregnancy-related symptoms often delaying diagnosis. In severe hypercalcemic crises, prompt parathyroidectomy is essential to resolve hypercalcemia and prevent organ damage.

In the lungs, hypercalcemia-induced deposition of calcium salts within alveolar spaces may impair gas exchange and contribute to the development of ARDS [4]. Hypercalcemia is known to affect vascular permeability and promote pulmonary edema, which may have further compounded respiratory failure in this case [5]. Autopsy cases have shown extensive calcium deposits in the alveoli, and it has been suggested that these deposits may have caused the lung damage, known as metastatic pulmonary calcification (MPC) [6]. Elevated calcium and phosphate levels play a critical role in the pathogenesis of MPC. In our case, the calcium-phosphate product on day 2 was 7.5 mmol^2^/L^2^, significantly surpassing the threshold (5.65 mmol^2^/L^2^) associated with MPC development [7]. In the present case, conventional CT revealed bilateral ground-glass opacities and consolidation, predominantly in the upper lobes, with septal thickening and minimal pleural effusion. However, conventional CT has limited sensitivity in detecting MPC caused by hypercalcemia [8]. Pulmonary edema related to hypercalcemia, with possible involvement of MPC, may have contributed to the respiratory failure. The mortality rate in cases of ARDS with hypercalcemic crisis is greater than 80% [4].

In the kidneys, hypercalcemia can cause nephrocalcinosis, characterized by calcium deposition in renal tubules [9]. This deposition can impair tubular function, reduce the eGFR, and precipitate AKI [10]. Renal replacement therapy using low-calcium or calcium-free dialysate is an effective approach to rapidly reduce serum calcium levels and mitigate morbidity and mortality [11]. However, calcium-free or low-calcium dialysate was not available in our case. In this patient, severe hypercalcemia likely accelerated renal damage, contributing to CKD still observed postoperatively. Her abdominal echocardiography after discharge showed hyperechoic areas suggestive of calcification in the renal medulla, a finding consistent with nephrocalcinosis [10].

In the present case, the resolution of respiratory and renal failure with hypercalcemia following parathyroidectomy underscores the critical importance of timely surgical intervention in severe cases of pHPT during pregnancy. Early diagnosis is essential for early intervention.

It is important to note that the definitive diagnosis of pHPT is confirmed by the presence of hypercalcemia along with a simultaneous elevation of PTH. However, parathyroidectomy was performed in this case without the PTH test result, as it was unavailable due to the New Year holidays. Japan has a longer New Year holiday period (over 5 days) compared to many other countries, which can lead to delays in medical services [12]. Japan's closure of businesses and diagnostic services for nearly a week is uncommon internationally. A system whereby laboratory tests can be performed during consecutive holidays in Japan is needed, for example, through setting up shifts. It is of great concern that delays in diagnosis can be fatal in conditions such as hypercalcemic crisis.

In our case, the patient developed hypercalcemic crisis during pregnancy. Pregnancy-associated changes in calcium metabolism, such as increased intestinal calcium absorption and decreased renal calcium excretion, may exacerbate hypercalcemia in women with pHPT [13, 14]. Potential contributors to the acute exacerbation of hypercalcemia include dehydration due to hyperemesis gravidarum and metabolic alkalosis secondary to vomiting, as observed in the present case.

This case report has some limitations. Biopsies of the lung and kidney were not performed, limiting the ability to confirm histopathological evidence of calcium deposition. A further limitation is the limited follow-up duration for long-term outcomes. Further studies and follow-up would help in understanding the long-term prognosis of multiorgan systems.

To the best of our knowledge, there have been no reported cases of pHPT during pregnancy leading to a hypercalcemic crisis to cause multiorgan failure. This case report contributes to the growing body of knowledge on hypercalcemic crisis and its effect during pregnancy, emphasizing the need for heightened clinical vigilance. The case report provides insights into hypercalcemia-induced organ dysfunction, including ARDS and nephrocalcinosis, which are poorly understood in the context of pregnancy. The importance of early intervention to prevent irreversible complications such as CKD is emphasized. Diagnostic delays, attributed to the prolonged New Year holidays in Japan, highlight critical issues concerning laboratory accessibility during extended holiday periods. Health policy implications are discussed, and better test availability during extended holidays to prevent delays of diagnosis and treatment of life-threatening conditions is advocated.

## Learning Points

- pHPT during pregnancy is rare but poses considerable risks, with overlapping symptoms from pregnancy-related conditions complicating timely diagnosis.
- Hypercalcemia during pregnancy can result in life-threatening complications both for mother and fetus, including ARDS and AKI.
- In severe cases of hypercalcemic crisis, timely surgical intervention (parathyroidectomy) is crucial for resolution of hypercalcemia and preventing further organ damage.
- The complex interplay between pHPT, pregnancy, and multiorgan dysfunction highlights the importance of a multidisciplinary approach for optimal maternal and fetal outcomes.

## Data Availability

Original data generated and analyzed during this study are included in this published article.

## Abbreviations

AKI: acute kidney injury
ARDS: acute respiratory distress syndrome
CKD: chronic kidney disease
CT: computed tomography
ECG: electrocardiogram
eGFR: estimated glomerular filtration rate
i-PTH: intact parathyroid hormone
IP: inorganic phosphate
MPC: metastatic pulmonary calcification
pHPT: primary hyperparathyroidism
99mTc-MIBI: 99mTc-methoxyisobutylisonitrile
V-V ECMO: veno-venous extracorporeal membrane oxygenation
